# Myiasis due to *Cordylobia anthropophaga*

**DOI:** 10.4269/ajtmh.19-0579

**Published:** 2020-02-05

**Authors:** Kosuke Yasukawa, Krishna Dass

**Affiliations:** 1 Division of Hospital Medicine, Department of Medicine, MedStar Washington Hospital Center, Washington, District of Columbia;; 2 Infectious Disease Consultant, MedStar Washington Hospital Center, Washington, District of Columbia

A 56-year-old man presented to the emergency department on the day of returning from Ethiopia because of multiple painful skin lesions. During his 2-month stay in Ethiopia, he visited a number of rural villages in Wolayta organizing public meetings. Approximately 1 week before returning to the United States, he noticed small raised red skin lesions in the left upper thigh, right lower abdomen, and lower back. In the emergency department, he was presumed to have multiple skin abscesses with cellulitis. He underwent a CT scan of the abdomen and pelvis with intravenous contrast to evaluate the extent of infection, which showed multiple skin thickening with inflammatory changes without fluid collection. He was started on intravenous vancomycin and piperacillin/tazobactam and was admitted to general medicine service for further intravenous antibiotic therapy.

When we evaluated the patient on the following day of admission, there were two erythematous skin lesions in his right lower abdomen and back with a part of larvae visualized in the center (Figure 1). For each lesion, pressure was applied from both sides and the larva was extracted using forceps. Two larvae were collected alive and sent for microscopic examination. Based on the shape and size, cuticular spines, and spiracular plates and peritremes, the larvae were most consistent with *Cordylobia anthropophaga* (tumbu fly) (Figure 2). There were two other ulcerative lesions, but larvae were not detected even with the use of bedside ultrasound. We suspected that the larvae had already migrated out from these lesions, although the patient had no recollection of seeing larvae.

**Figure 1.**
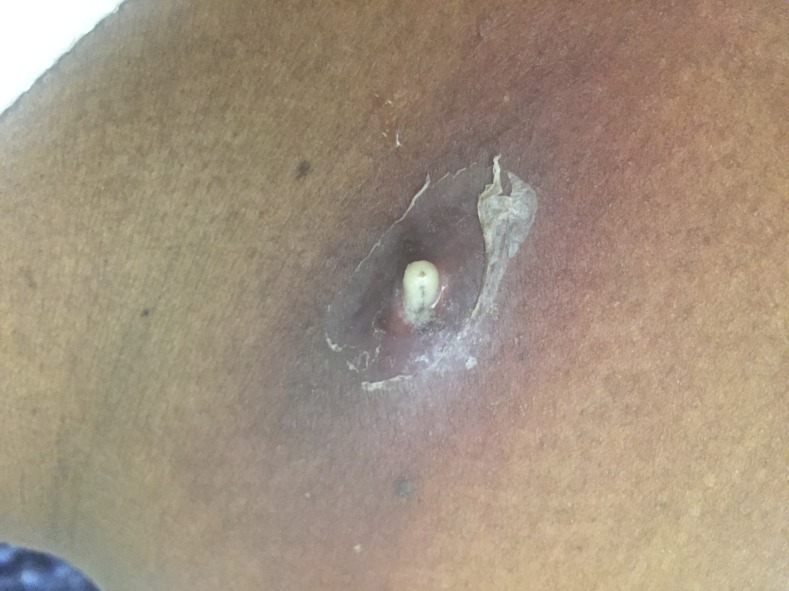
Skin lesion with a partly visualized larva. This figure appears in color at www.ajtmh.org.

**Figure 2.**
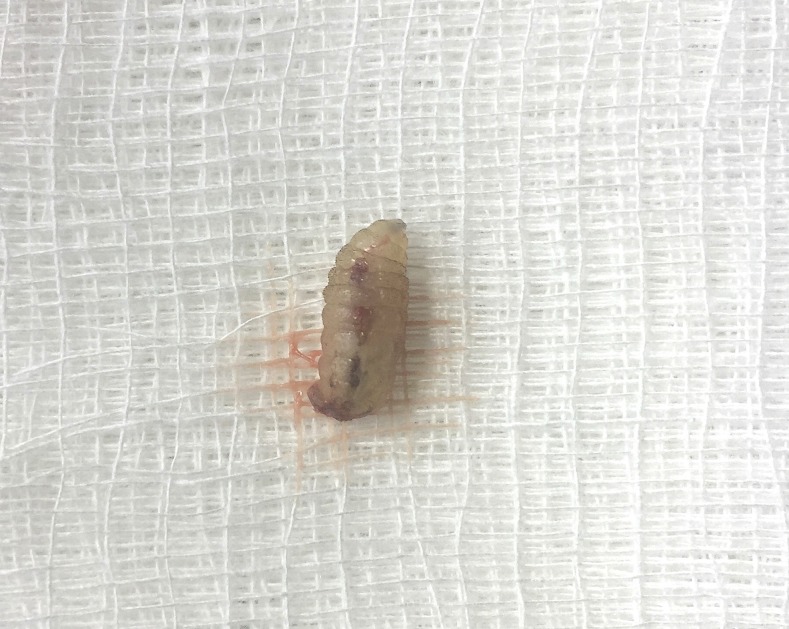
*Cordylobia anthropophaga* larva. This figure appears in color at www.ajtmh.org.

Cases of myiasis due to *C. anthropophaga* have been reported in the Unites States in patients returning from endemic areas.^1^ Adult flies tend to lay eggs on soiled clothing, and the larvae burrow into the subcutaneous tissue after contact with the skin.^2^ Careful examination of all skin lesions and the knowledge of myiasis are important for the diagnosis and to avoid unnecessary testing and antibiotic administration. Ultrasound can be used when direct visualization is difficult.^3^ Various techniques have been proposed to remove the larva, including mechanical removal, forcing the larvae to the surface by occlusion of the punctum, and hydraulic expulsion.^3,4^

## References

[1] Rice PL Gleason N, 1972 Two cases of myiasis in the United States by the African tumbu fly, *Cordylobia anthropophaga* (Diptera, Calliphoridae). Am J Trop Med Hyg 21: 62–65. 5007189 10.4269/ajtmh.1972.21.62

[2] Park JJ Costello J, 2017 Tumbu fly larvae. N Engl J Med 376: e22. 28355510 10.1056/NEJMicm1609697

[3] Francesconi F Lupi O, 2012 Myiasis. Clin Microbiol Rev 25: 79–105. 22232372 10.1128/CMR.00010-11 PMC3255963

[4] Dybbro E Fordyce P Ponte M Arron ST, 2014 Hydraulic expulsion of tumbu fly larvae. JAMA Dermatol 150: 791–792. 24647598 10.1001/jamadermatol.2013.9571

